# Passivation Species Suppress Atom-by-Atom Wear of Microcrystalline Diamond

**DOI:** 10.1021/acsami.5c08647

**Published:** 2025-09-18

**Authors:** C. Leriche, E. Pedretti, O. Sahin, D. Kang, M. C. Righi, B. Weber

**Affiliations:** † 530573 Advanced Research Center for Nanolithography, Science Park 106, 1098 XG Amsterdam, The Netherlands; ‡ Van der Waals−Zeeman Institute, Institute of Physics, University of Amsterdam, Science Park 904, 1098 XH Amsterdam, The Netherlands; § Department of Physics and Astronomy “Augusto Righi”, 9296 University of Bologna, Via Zamboni 33, 40126 Bologna, Italy

**Keywords:** tribochemical wear, passivation species, plowing friction, nonrepeated wear, diamond, multi-asperity contact

## Abstract

Despite its supreme hardness, (synthetic) diamond wears. Due to the small volume loss involved, diamond wear is challenging to quantify, specifically for multicontact interfaces. Consequently, identifying which wear mechanisms dominate the degradation of macroscopically loaded diamond interfaces has remained an open challenge. Using a topography difference method based on atomic force microscopy imaging, we observe the wear of multi-asperity microcrystalline diamond (MCD) surfaces sliding nonrepeatedly against silicon nitride (Si_3_N_4_)-coated silicon wafers. By examining the wear scars on Si_3_N_4_, which can be seen as footprints of the MCD surface, we are uniquely able to track the nanoscale wear of individual MCD crystallites. Our MCD wear measurements show that the diamond wears atom-by-atom and that this wear is accelerated in the absence of passivation species in the environment. This conclusion is confirmed by ab initio molecular dynamics simulations, highlighting how diamond surface passivation suppresses interfacial bonding. Our results thus demonstrate that atom-by-atom wear occurs even in realistic, multi-asperity diamond contacts and that environmental passivation provides a practical and effective means to control interfacial degradation in advanced tribological systems.

## Graphical abstract

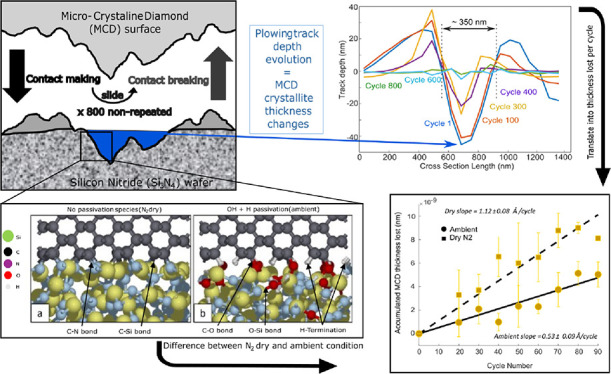

## Introduction

Surfaces subject to contact and friction break down and need replacement: the wear of materials has been estimated to cost 4% of the gross domestic product in industrialized nations.^1–3^ Synthetic diamond coatings have attracted significant attention because such coatings can offer high wear resistance under harsh contact conditions,^4^ making it possible for applications subject to tribological contact to last longer before requiring replacement. For example, synthetic diamond has found application in cutting tools, drills, bearings, and microelectromechanical systems (MEMS).^5–9^ Arguably, the most stringent requirements in terms of wear can be found in the semiconductor industry, where nanometer-scale patterning requires subnanometer-scale control over the positioning of, i.e., silicon (Si) wafers. This means that the smallest amounts of wear can already be significant, necessitating an understanding of the diamond wear process down to the smallest of length scales.

The mechanisms behind the nanoscale wear of diamond are debated. Both computer simulations^10–12^ and atomic force microscopy (AFM) experiments^13–15^ have demonstrated that diamond surfaces can wear in an atom-by-atom wear process.^16–21^ In this atomic attrition wear, covalent bonds are formed between the carbon atoms on the diamond surface and, i.e., silicon or oxygen atoms on the counter surface as a first step toward carbon removal.^22,23^ Theoretical studies have indicated that the diamond C(110) surface, specifically, is susceptible to tribochemical wear of zigzag carbon chains,^11^ while the wear can be suppressed through surface passivation,^24^ for example, with hydrogen^25,26^ which then blocks the bond formation with the counter surface. Furthermore, graphitization and amorphization^27–30^ of the topmost diamond have been reported.

As an alternative to the atomic attrition of the topmost diamond, it has been suggested that diamond wear may dominantly take place through a fracture process^31–33^ in which cracks propagate into the diamond matrix such that clusters of carbon atoms—or complete crystallites—can be removed from the diamond surface.^34,35^ In single asperity AFM experiments with high contact pressures, such fracture and detachment of atom clusters have been observed.^15,34,36^ Furthermore, multiasperity contacts can contain even larger contact patches, thereby surpassing the critical scale for adhesive wear and promoting crack formation.^32^

The observation and prediction of these differing diamond wear mechanisms raise a key question for the application of diamond in larger contacts: how will industrially representative diamond multicontacts wear? While fracture-induced wear mechanisms are thought to become more dominant as contacts scale up,^32,35^ such large scales remain challenging to investigate with accurate simulations in which the system size is limited. Furthermore, it is highly challenging experimentally to characterize and quantify the wear^37^ of diamond multicontacts, because the wear volumes are orders of magnitude smaller than the size of the system. Consequently, there remains a large gap between the theoretical understanding of diamond wear at the atomic scale and the experimental observations of diamond wear in multiasperity contacts.

To address these challenges and to link all the way from fundamental insights at the atomic scale to industrially relevant multicontact^38^ interfaces, we investigate the wear behavior of microcrystalline diamond (MCD) coatings interfaced with Si_3_N_4_ wafers. Through a combination of ab initio molecular dynamics (AIMD) and unique, precision wear experiments supplemented by ex-situ AFM characterization,^39^ we show that diamond multi-contacts wear through atomic attrition in which the presence of passivation species plays a key role.

## Experimental and Computational Methods

Silicon carbide (SiC) spheres (DitHolland, 3 mm diameter) were coated with a 1 μm thick MCD layer (Fraunhofer Institute for Surface Engineering and Thin Films). Si wafers (University wafer) coated with a 100 nm thick Si_3_N_4_ layer (amorphous, grown with the LPCVD method) were used as the counter surface. The mechanical properties of Si_3_N_4_ and MCD coatings are reported in Table 1.

**Table 1:** Material Properties of the Si_3_N_4_ Wafer (Measured by Nanoindentation) and the MCD Coating.^8^

materials	Young’s modulus (GPa)	Poisson’s ratio	hardness (GPa)
Si_3_N_4_ wafer	270 ± 12	0.17	22.3 ± 1.5
MCD coating	1000 ± 200	0.07	90 ± 10

The diamond-coated hemisphere is immobilized onto a nanoindenter (FT-I04, Femtotools, see SI for more details) to perform the nonrepeated^40^ wear experiment, in which each stroke takes place on a previously untouched region on the wafer (Figure 1). Our choice for nonrepeated wear is motivated by precision positioning applications in which wear-resistant materials are repeatedly contacted by fresh samples. The friction/wear experiment consists of 800 strokes of 20 μm sliding distance with a 70 mN load applied and an imposed sliding velocity of 2 μm/*s*. During each sliding cycle, the friction force is measured.

**Figure 1:**
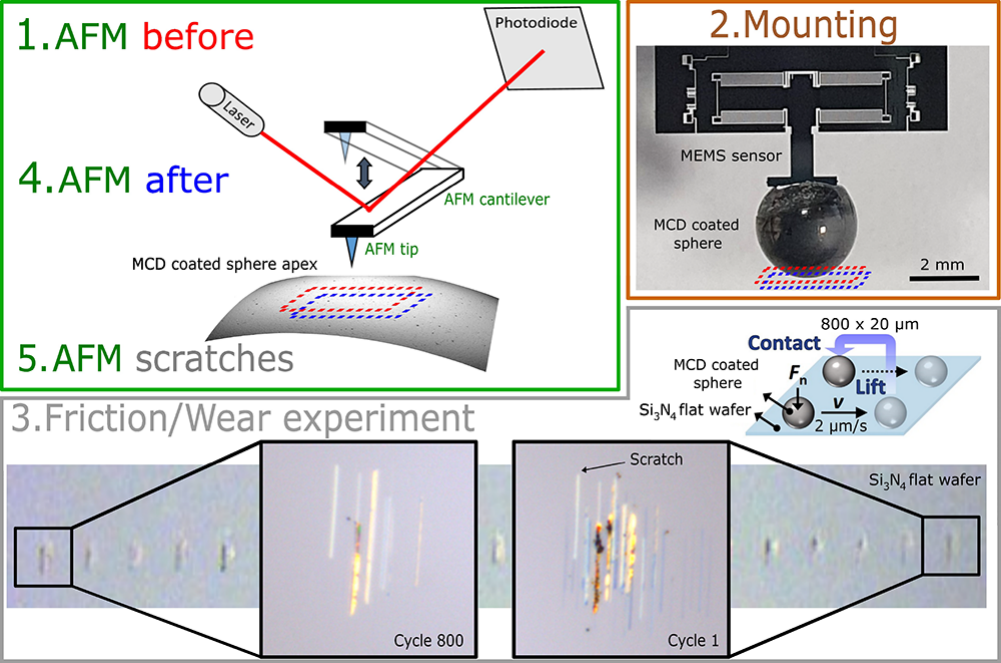
Imaging and friction/wear experiment. Green frame: we use atomic force microscopy (AFM) to image the MCD surface before and after the wear experiment as well as the scratches made on the Si_3_N_4_ wafer by the MCD coating during the friction/wear experiment. Brown frame: Photo of a SiC hemisphere coated with MCD and glued onto a MEMS sensor for the friction/wear experiment. The dashed blue and red rectangles in the green and brown frames indicate that AFM measurements on the hemisphere are centered on the apex that contacts the flat Si_3_N_4_ in the friction/wear experiment. Gray frame: A schematic representation of the nonrepeated wear experiment is depicted in the top-right corner of the frame. Optical microscopy images of the scratches made by the MCD coating on the Si_3_N_4_ flat indicate that as the experiment progresses, fewer scratches are visible.

For imaging the MCD surface before and after the wear experiments as well as the scratches on the Si_3_N_4_ flat, an AFM (Dimension ICON, Bruker) was used in the tapping mode^41^ in an ambient environment.

For the computational study, the choice of using DFT over classical force fields stems from the fact that tribochemical processes involve bond breaking and forming, for which an accurate quantum-mechanical description of the electronic behavior of the system is paramount.

We approached the study in two steps. First, by means of AIMD simulations, we studied the dependence of diamond surface reactivity on the passivating species since the initial step of tribochemical wear necessarily involves the formation of chemical bonds across the diamond-Si_3_N_4_ interface. In the second step, through ab initio static calculations, we studied how likely certain bonding configurations between Si_3_N_4_ clusters and diamond are to cause atomistic wear by detaching carbon atoms from the surface.

In the AIMD simulations, the Si_3_N_4_-diamond interface was created using two slabs (periodic in the *x−y* plane), placing the diamond slab on top of the Si_3_N_4_ slab. The diamond slab consisted of 6 × 4 cells of the C(110) surface, with 7 carbon layers, and a lateral size of 15.13 × 14.27 Å. The amorphous Si_3_N_4_ slab was ≈12 Å thick, with the same lateral size as the diamond slab, and contained 227 atoms. A vacuum region of more than 10 Å was included above the diamond slab in the *z*-direction to suppress spurious interactions with periodic replicas. Due to the large size of the cells, it was sufficient to sample the Brillouin zone at the Γ-point. It is worth mentioning that the simulated systems contained up to 670 atoms, which is a considerable size in the context of AIMD simulations.

The tribological conditions of sliding under load were realized by imposing on the topmost diamond layer a normal load of 23 GPa, corresponding to the harsh conditions at which plowing friction occurs when the load exceeds the hardness of the Si_3_N_4_ substrate, and a constant speed in the *x*-direction to reproduce sliding. The positions of the atoms in a 4.5 Å-thick slice at the bottom of the Si_3_N_4_ slab were kept fixed to counterbalance the applied load. More details on the computational setup are reported in the Supporting Information.

## Results and Discussion

### Diamond Wear Experiment

Figure 2a shows the MCD surface before the wear experiment that was conducted in an ambient environment. We zoomed-in to a single MCD asperity or crystallite and quantified the wear of that crystallite (Figure 2b). By plotting the height profile across the MCD crystallite before and after the wear experiment, we can quantify how the MCD surface changes during the wear experiment. Focusing on this wear of individual diamond crystallites, we see flattening of the sharp crystallite surface and can quantify the local height loss of up to 100 nm. Interestingly, considering that this height loss may take place gradually during 800 strokes, this result is consistent with an atom-by-atom^16,18,19^ wear process, as only a single atomic layer would be removed from the diamond per cycle (20 μm of sliding).

**Figure 2:**
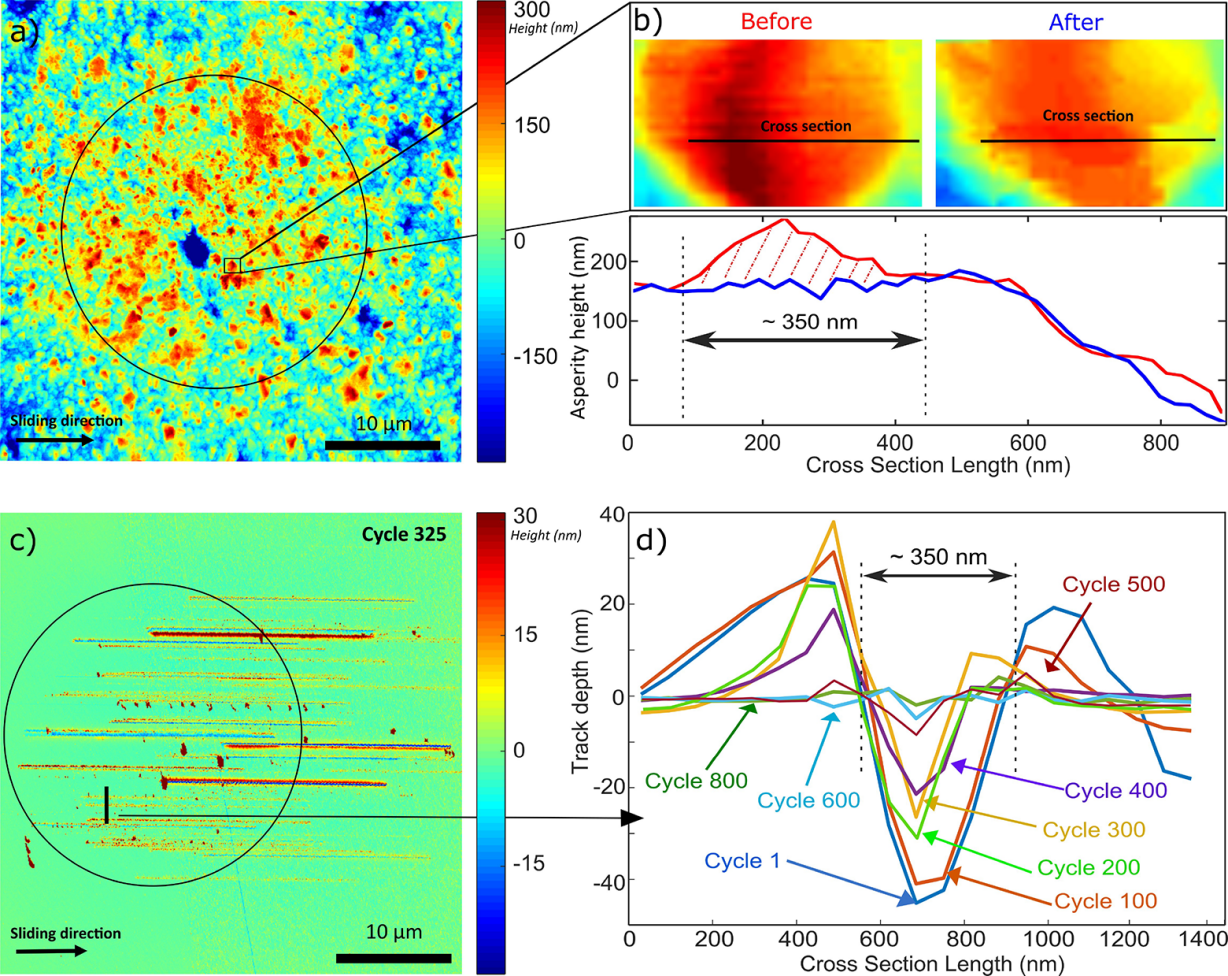
(a) AFM image of the MCD surface measured before the wear experiment in an ambient environment. The black circle represents the Hertzian contact area. (b) Zoom-in view of an MCD crystallite (top box) and plot of a height profile across the crystallite (bottom box) measured before (red) and after (blue) the wear experiment. More before and after images of the spheres are shown in Figure S3. (c) Scratches made by the MCD coating on the Si_3_N_4_ wafer in cycle 325, imaged by AFM (for more examples, see Figures S4 and S5). The circle represents the Hertzian contact area (average Hertzian pressure of 0.2 GPa) and encloses the starting point of all scratches. The contact pressure within the plowing asperity contacts is expected to be equal to the Si_3_N_4_ hardness of approximately 23 GPa. (d) Cross sections of the scratch indicated in (c), measured at various cycles of the wear experiment. The depth of the scratch and the pile-up of Si_3_N_4_ next to the scratch decrease as the experiment progresses, indicating that the diamond crystallite causing the scratch is wearing.

### AFM Imaging of Scratches

To get a better understanding of the evolution of the MCD surface throughout the experiment, we image the scratches left on the Si_3_N_4_ surface by the MCD. We want to emphasize that although Si_3_N_4_ wear is a relevant topic on its own,^42^ the main purpose behind imaging the scratches on the Si_3_N_4_ in this study is to obtain a quantitative understanding of the MCD wear. As shown in Figure 2c, the hard MCD crystallites plow^43,44^ through the softer Si_3_N_4_. Impressively, each cycle in the wear experiment results in a cluster of scratches on the Si_3_N_4_ surface that can be located and imaged as each sliding cycle is performed on a previously untouched part of the Si_3_N_4_ flat. The nonrepeated nature of the wear experiment thus results in scratches on the Si_3_N_4_ that can be linked to cycle numbers in the wear experiment. We interpret the scratches on Si_3_N_4_ as footprints of the diamond crystallites that cause the scratches. On and next to the scratches, we can also observe a significant amount of piled-up Si_3_N_4_ as well as Si_3_N_4_ particles. For the deepest scratches, the amount of piled-up Si_3_N_4_ is maximal, and the displaced Si_3_N_4_ partially covers the scratches, making measurement of the depth of the scratches challenging. To study the evolution of the depth of the scratches on Si_3_N_4_, we, therefore, select those scratches that are most clearly visible (Figure 2c).

In Figure 2d, we plot and align height profiles taken perpendicular to the sliding direction for the same scratch identified in subsequent cycles during the friction/wear experiment. This enables us to observe a decrease in the depth of the scratch as the cycle number increases. Importantly, this result supports the previous conjecture that the MCD crystallites wear gradually, although some variation in the rate at which the scratch depth decreases over the course of the experiment is visible. Figure 2d also shows that the cross-sectional area of the piled-up material on both sides of the scratch is approximately equal to the cross-sectional area of the scratch itself, indicating that the pile-up material is Si_3_N_4_. We thus argue that the decrease in the depth of the Si_3_N_4_ scratch indirectly reveals the gradual smoothening of the MCD crystallites plowing through the Si_3_N_4_ wafer in each stroke.

### Quantifying Diamond Wear

To translate the evolving scratch profile from Figure 2d into a diamond wear rate,^19,43,44^ we assume that the diamond crystallites causing the scratches are cone-shaped. We then calculate the volume of these cone-shaped MCD crystallites for various cycles during the wear experiment based on the cross-sectional profiles of the scratches on the Si_3_N_4_. To obtain the MCD wear volume, we subtract the crystallite volumes calculated based on scratches that were made subsequent to one another. Furthermore, by dividing the resulting wear volume by the (average) area over which the MCD crystallite contacts the Si_3_N_4_ wafer, we obtain the average thickness of MCD that is worn during the cycles that are covered by the calculation. For each environmental condition, we perform this analysis on two different Si_3_N_4_ scratches, selected based on the absence of substantial debris covering the scratches (Figures S4 and S5). These two scratches thus relate to two different MCD crystallites. We analyze each scratch based on three height profiles taken orthogonal to the sliding direction at different locations along the scratch. This analysis enables us to plot (Figure 3) the cumulative diamond thickness lost for an experiment performed in a dry nitrogen environment (RH < 5%, squares) as well as an experiment performed in an ambient environment (RH = 50%, circles). This analysis uniquely provides nanometer-scale insight into the diamond wear behavior throughout the 800 sliding cycles in the experiment.

**Figure 3:**
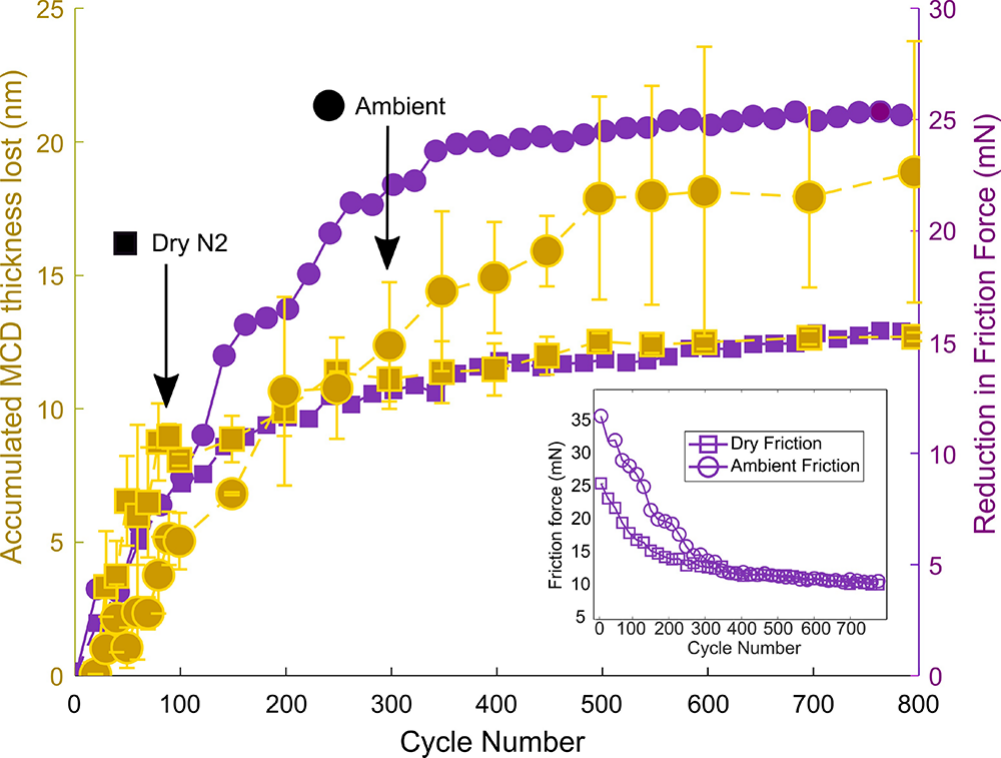
Accumulated diamond thickness lost and friction reduction versus cycle number. For both ambient and dry environments, the wear is strongly correlated to the change in friction. Inset: friction evolution of the system during the full experiment.

The Si_3_N_4_ scratches are generated as long as the diamond crystallites plow through the Si_3_N_4_ wafer, exerting a pressure equal to the Si_3_N_4_ hardness. We, therefore, expect that the diamond wear rate, expressed as diamond volume lost per unit contact area per unit sliding distance, remains constant until the diamond asperities become smooth enough so as not to plow through the Si_3_N_4_ anymore. Indeed, we see that the cumulative diamond thickness lost for both the ambient and the dry nitrogen experiments increases at a nearly constant rate, after which a plateau value is reached after cycle 500 and cycle 200, respectively, for the experiments conducted in ambient and dry nitrogen environments. The MCD-coated hemisphere that was used in the ambient environment experiment made, on average, deeper scratches on the Si_3_N_4_ wafer, translating into higher plowing friction and more total diamond thickness lost (Figures S7 and S8). This difference between the diamond spheres leads to a crossover in the accumulated MCD thickness lost after about 200 cycles (Figure 3). We observe that for the first 90 cycles—where both the ambient and dry experiments display constant diamond wear rate—the cumulative diamond wear increases faster in the dry conditions than in the ambient environment.

Additional insight into the wear behavior is obtained by monitoring the dynamic friction force during wear experiments. As a pristine section on the Si_3_N_4_ flat is visited in each stroke, any changes in friction during the wear experiment are caused by changes to the MCD-coated hemisphere. In Figure 3, we plot the reduction of the friction force with respect to its initial value alongside the wear data. Interestingly, the friction and the cumulative diamond thickness lost correlate well, supporting our wear analysis.

The observations described above confirm that plowing friction^45^ dominates the tribological system; when the scratches on the Si_3_N_4_ wafer become too shallow to be measured, the friction force stabilizes. Friction and wear correlate well for both the experiment conducted in an ambient environment and the experiment conducted in dry N_2_; the deeper scratches observed in the ambient environment experiment lead to higher initial friction and longer continuation of the steady-state wear process, compared to the experiment conducted in a dry nitrogen environment (more details in Supporting Information). A rough estimate of the plowing friction force can be obtained by multiplying the plowing cross section by the Si_3_N_4_ hardness. Assuming 30 scratches of width 300 nm and depth 50 nm (Figures 2, 4 and Supporting Information), we get to a plowing force of 5 mN; the same order of magnitude as the observed reduction in friction in Figure 3. The good correlation of the wear and friction during run-in^15,26,34,46^ suggests that the friction signal can provide in situ information on the wear-induced evolution of the diamond topography. Indeed, the observed diamond run-in behavior, dominated by changes in interface topography, is reminiscent of that observed previously in various experiments.^31,47,48^

**Figure 4:**
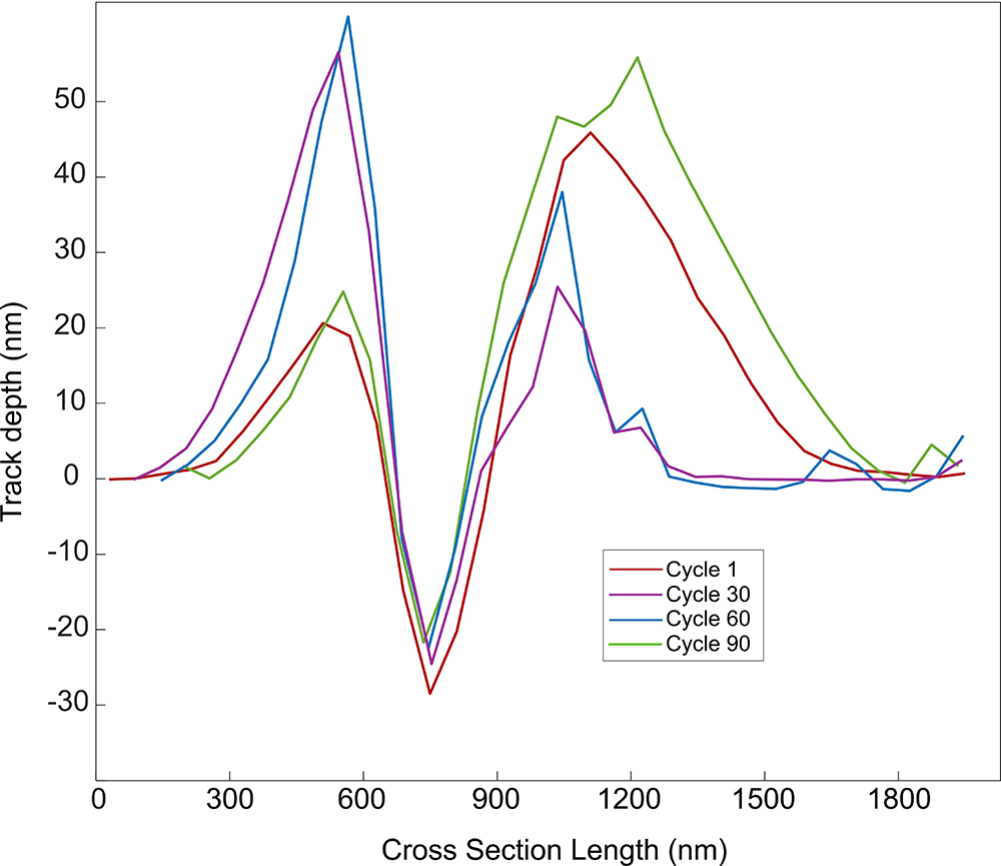
Evolution of the cross section of a scratch in the first 90 cycles of the ambient wear experiment.

### Atom-by-Atom Wear

We now consider only the first 90 cycles of the wear experiment (Figures 4 and 5), in which a large number of diamond crystallites plow through Si_3_N_4_ for both the ambient and dry nitrogen experiments. We observe that, within the contact area, the cumulative thickness of diamond that gets worn off the surface scales approximately linearly with sliding distance. As the contact pressure within the plowing contacts should be constant, this result also indicates that wear scales with applied normal force. The wear rates we observe are very low, especially compared to those associated with atom cluster removal or plasticity.^49,50^ Our analysis demonstrates that the amount of wear per cycle is consistent with an atom-by-atom wear process. A linear fit was applied to the data, which impressively shows diamond wear rates of only 1.12 ± 0.08 Å/cycle for the dry nitrogen experiment and 0.53 ± 0.09 Å/cycle for the ambient environment experiment. We found a factor 2 difference in the MCD wear rate depending on the availability of environmental passivation species. In our experiment, changing the relative humidity changes the thickness of the water layer present on both the MCD and the Si_3_N_4_ surfaces. Therefore, the reduction in relative humidity may significantly impact the coverage of passivating O, H, and OH species at the interface, as previously suggested by both experimental^26,51^ and theoretical^52,53^ studies. In order to provide an atomistic understanding of the interplay between environment and wear rate, we conduct first-principles density functional theory (DFT)^54^ calculations that address the same system under the same contact conditions.

**Figure 5:**
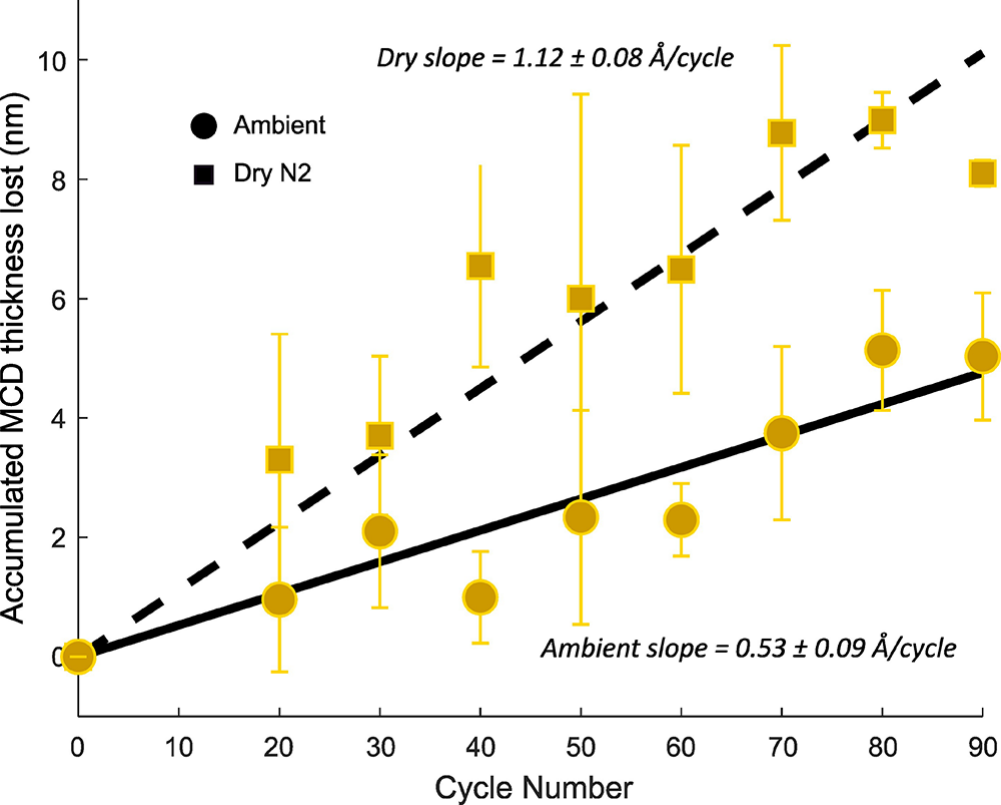
Diamond wear in the first 90 cycles of the wear experiment. The data are fitted with a linear function crossing the origin of the plot. Error bars represent the variation in diamond thickness lost found for two independent crystallites per environmental condition. For each crystallite/scratch, three cross sections along the scratch were used. The reported slope fits include 95% confidence intervals. More examples of evolving scratches are reported in Figures S4–S8.

### Molecular Dynamics Simulations

Due to the vastly different time scales between AIMD and experiment, our simulations target the chemistry underlying the observed wear rates. Rather than reproducing wear depth, we focus on how passivating groups influence bond formation at the diamond–Si_3_N_4_ interface, providing mechanistic insight into the experimentally observed suppression of wear under ambient conditions. In the AIMD simulations, we impose an interfacial normal stress of 23 GPa, set by the hardness of Si_3_N_4_ (Table 1). This choice is motivated by the experimental observation of plowing friction, which suggests that those diamond crystallites that indent and scratch the Si_3_N_4_ experience a normal interfacial stress equal to the hardness of Si_3_N_4_ (Figure S2). The results of our AIMD simulations (more details in the Methods and Supporting Information) for the variously terminated C(110) surface sliding against Si_3_N_4_ in different directions are presented in Figure 6. Just from visual inspection, it is already clear that the presence of passivating species strongly reduces the formation of bonds between carbon atoms of diamond and Si/N atoms of the Si_3_N_4_: in the simulation without passivation species, representative of the dry N_2_ environment, all surface carbon atoms are chemically bonded to Si_3_N_4_, while the H+OH passivation, representative of the humid environment, visibly reduces the formation of C–N and C–Si bonds. Some bonds at the interface are still formed, mainly at the locations of hydroxyl groups, which form Si–O–C bonds after the transfer of their H atoms to Si_3_N_4_. Moreover, as visible in Figure 6b,e, a few oxygen atoms themselves are transferred from diamond to Si_3_N_4_ and slowly migrate into the Si_3_N_4_ bulk, exposing the diamond surface to direct bonding with Si_3_N_4_. On the other hand, hydrogen seems to be the most effective in hindering chemical bonding:^55^ the H-passivated sites in the H+OH passivated surface are not involved in bonding throughout the entire simulation. This behavior is clearly demonstrated by the fully H-passivated diamond surface, which shows no bond formation, even when subjected to 23 GPa of contact pressure due to Pauli repulsion. We emphasize that H passivation is very stable under extreme conditions with no dissociation after 20 ps of sliding.

**Figure 6:**
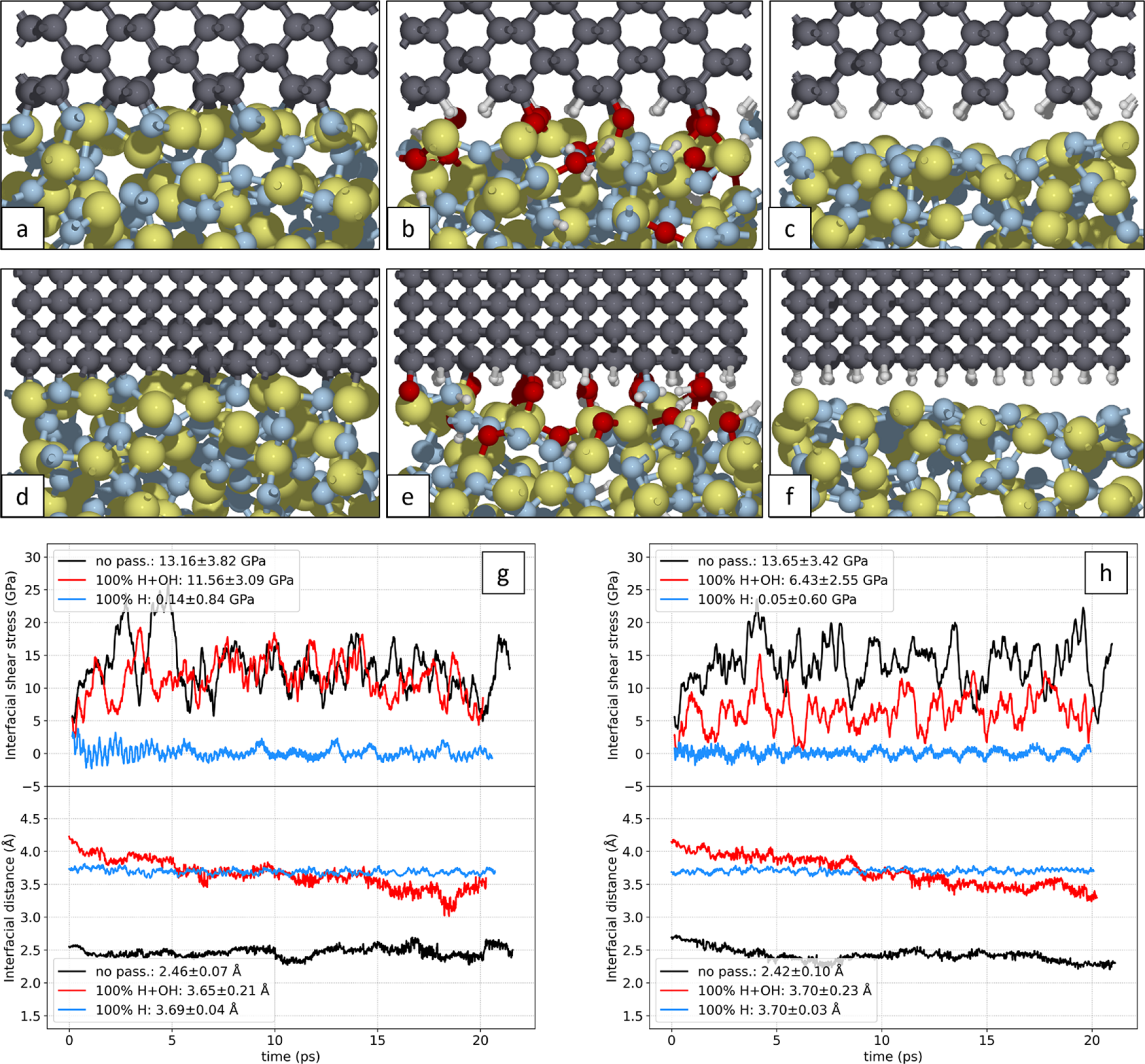
Snapshots of the C(110)—amorphous Si_3_N_4_ interface after 20 ps of AIMD simulation of sliding under applied contact pressure of 23 GPa. (a–c) refer to the sliding in the direction perpendicular to the zigzag chains of the C(110) surface for the different passivating species: no passivation, 100% H–OH passivation, and 100%H passivation of the diamond surface, respectively. (d–f) refer to sliding in the direction of the zigzag chains, for the same passivating species. Panels (g, h) show the interfacial shear stress (friction force per unit area) and the interfacial distance between diamond and Si_3_N_4_ for the sliding direction perpendicular and parallel to the zigzag chains, respectively. The color code of the atoms is as follows: gray for C, yellow for Si, blue for N, red for O, and white for H.

To make the comparison of various diamond surface terminations more quantitative, we analyze the simulation trajectories by calculating the interfacial distance between the diamond surface and Si_3_N_4_, obtained as the difference between the average z-coordinates of the interfacial C atoms (upper slab) and Si/N atoms (lower slab). Furthermore, we calculate the friction force as the force opposing the sliding motion, measured as the sum of the x-component of the force on the topmost diamond layer, where the external load and sliding velocity constraints are applied. To make the graphs more readable, we filter fast oscillations in the forces with a moving average over 200 time steps. We then divide the forces by the area of the cell to obtain the interfacial shear stress (friction force per unit area). The trends of interfacial shear stress and sliding distance along the trajectories are reported in Figure 6g,f, for the perpendicular and parallel sliding directions, respectively, comparing the three different types of passivation. The nonpassivated surface (dry environment) shows the highest friction and lowest interfacial distance, due to the full chemical bonding at the interface. The H+OH passivation reduces the shear stress (by a small amount, 13.2 to 11.6 GPa, for the perpendicular direction and much more, 13.7 to 6.4 GPa, in the parallel sliding direction) and increases the interfacial distance by about 50% for both directions. The H-passivated surface shows a dramatic reduction in interfacial shear stress, since no chemical bonds need to be broken to enable slip; the friction force is only caused by Pauli repulsion between the hydrogen atoms on the diamond and the Si_3_N_4_ surface, which deforms under the diamond slab during sliding. For hydrogen-passivated diamond, the interfacial distance to the Si_3_N_4_ is larger than for the nonpassivated diamond, and it is similar to the interfacial distance observed for the H+OH passivation case. As the simulation progresses, the H-passivated surface maintains a constant interfacial distance, while the H+OH-passivated surface gets closer to the Si_3_N_4_ as the sliding progresses due to the loss of some OH groups. Indeed, the gradual replacement of OH passivation with H-passivation may occur in our wear experiments, as the experimental interfacial shear stresses are below 3.3 GPa, based on an upper limit for the normal stress of 23 GPa and an upper limit of the adhesive friction coefficient (in the absence of plowing) of 0.14 (Figure 3). These relatively low shear stresses and friction coefficients favor mild, atom-by-atom wear mechanisms over more severe atom cluster removal or plasticity.^32^ A limitation in the comparison between experiment and simulation is that in the experiment, various diamond crystal faces interact with the Si_3_N_4_ counter surface, while the presented simulations probe the reactive C(110) surface.

The overall result, which emerges from this comparative analysis, is that passivating species coming from a humid environment are effective in protecting the diamond surface and preventing bonding to the Si_3_N_4_, which is the first step that enables atom-by-atom wear. Hydrogen, which is present only in water but not in dry N_2_, is more effective than oxygen in passivating the diamond surface and suppressing interfacial bonding. This picture aligns qualitatively with our experimental findings. An increased interfacial distance due to passivation species (Figure 6g,h) bottom graphs) leads to fewer interfacial chemical bonds, thereby reducing atom-by-atom wear, as observed in our experiments.

To probe diamond wear more directly in the calculations, we evaluate the ability of various small Si_3_N_4_ clusters to pull carbon atoms out of the diamond matrix, starting from an initial bonding configuration (Figure 7). Among the Si_3_N_4_ clusters that we consider, the bidentate configuration, with two N atoms bonded to a carbon atom, is most effective in creating diamond wear. In the calculations, the central Si atom is in a tetrahedral configuration and is bonded to four N atoms, as in Si_3_N_4_, while N atoms are passivated with hydrogen to saturate dangling bonds. By vertically pulling the cluster from the central Si atom, we observe an initial small displacement of the bonded carbon atom of the zigzag chain of the C(110) surface. Increasing displacement of the cluster then results in progressive detachment of the whole zigzag carbon chain. The bond-breaking steps are reported in Figure 7, along with the energy variation as a function of displacement and the residual force on the Si atom at each step, which corresponds to the pulling force necessary to maintain the vertical position in each configuration. A force of ≈0.6 nN is reached before each carbon detachment, after which the force drops to a value close to zero, rising again as the next detachment approaches. This wear behavior of chain detachment initiated by bonds in a bidentate configuration is consistent with previous findings for silica^56,25^ and proves that chemical bonding can cause atomic-level wear of diamond, implying that the presence of passivating species that hinder bond formation can be effective in suppressing these atomic-scale wear processes.

**Figure 7:**
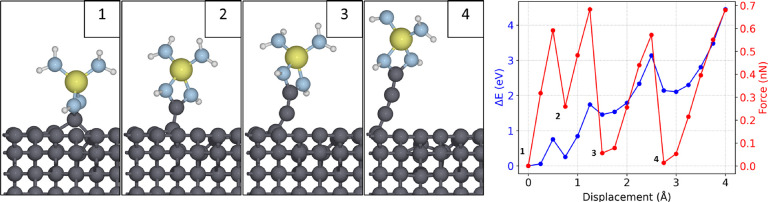
Wear mechanism of the C(110) surface by a small Si_3_N_4_ cluster initially bonded in a bidentate configuration. Starting from the initial configuration (1), the cluster is progressively pulled vertically by displacing the z coordinate of the central Si atom, measuring the force and the energy variation with respect to the starting point while the cluster is being pulled. Peaks in the force are followed by sudden drops (numbers 2, 3, and 4 in the graph) corresponding to the detachment of a carbon atom from the zigzag surface chain.

## Discussion and Conclusions

In summary, we studied MCD-on-Si_3_N_4_ friction and wear and found a low wear rate (Å height loss per 20 μm sliding distance) of individual MCD crystallites within multi-asperity interfaces under harsh conditions, consistent with atom-by-atom wear of the MCD. It is important to note that while our AFM characterization of scratch profiles provides very detailed insight into the multi-asperity wear behavior, we cannot experimentally exclude the possibility that diamond wear events may involve larger clusters of atoms.^49,50^ Based on our data, an upper limit for the thickness of such clusters would be a few nanometers (Figures 4, 5 and S6). Our experiments represent a worst case scenario in terms of MCD wear because the high hardness of the Si_3_N_4_ wafer combined with the roughness of the MCD surface leads to contact pressures up to the hardness of Si_3_N_4_: 23 GPa. Indeed, silicon is known to display atom-by-atom wear at contact pressures equal to 20% of its hardness.^16^ For the diamond contacts in this study, 23 GPa of normal stress corresponds to a similar fraction of the diamond hardness, suggesting that atom-by-atom wear is a likely scenario. The friction reduction in our study is strongly correlated with the wear of the MCD, highlighting that plowing gradually decreases as the MCD crystallites wear, such that the measured plowing friction forces indirectly provide information on the wear of the MCD. The present study is limited in the sense that we report in detail only on two independent experiments. However, more diamond-on-Si_3_N_4_ experiments have been conducted (see Supporting Information), confirming that the friction behavior is repeatable, and we will report on the influence of stroke length on the wear behavior in a future publication. Opposed to the constant (averaged) wear rate in the first 90 cycles of our experiments (Figure 5), we see some variation in the wear rate of an individual crystallite over the course of the full 800 cycle experiments (Figure 2d). We hypothesize that on these longer experimental time scales, diamond wear, variations in ball-on-flat alignment, and release of particles during plowing may influence the precise normal force distribution among the diamond crystallites, leading to complex asperity interactions;^42^ these effects could be studied in more detail in the future. Immersing the system in dry nitrogen to reduce the presence of passivating species, the wear of MCD accelerates by almost a factor 2 compared to the wear observed in ambient conditions; MCD wear increases from 0.53 ± 0.09 Å/cycle in ambient to 1.12 ± 0.08 Å/cycle in dry nitrogen. This result is qualitatively consistent with the known impact of environment on diamond friction.^57^ To quantitatively place our wear rates in perspective, we translate into the unit mm^3^/Nm and compare to the nonrepeated wear of other material combinations in Table 2.

**Table 2:** Approximate Nonrepeated Wear Rates of the Harder (Left) Material in Ambient Environment,^40,42,58^ Including the Present Result for MCD

materials	Si–Si	Si_3_N_4_–Si	SiC–Si	sapphire-Si	MCD-Si_3_N_4_
wear rate (mm^3^/Nm)	5.2 × 10^–4^	1 × 10^–4^	7.7 × 10^–6^	6.5 × 10^–6^	1.2 × 10^–7^

Furthermore, our results can be compared to previously measured diamond wear rates in reciprocal sliding experiments. At the macroscale, in DLC-on-Si_3_N_4_ sliding wear experiments, DLC wear rates ranging from 10^–8^–10^–7^ mm^3^/Nm have been reported.^59–61^ As MCD is harder than DLC, and therefore more difficult to wear, this comparison suggests that the nonrepeated nature of our experiments leads to higher wear rates, likely through the contact making-breaking steps and the reduced presence of third bodies.^40^ At the nanoscale, higher wear rates for DLC and diamond-coated AFM probes sliding against silicon or Si_3_N_4_ are reported,^13,15^ ranging from 10^–7^–10^–6^ mm^3^/Nm. Our macroscale experiments result in a lower wear rate for MCD compared to nanoscale wear tests in the AFM. This may be attributed to the observation of fracture wear in AFM experiments due to the extreme aspect ratio of AFM probes, in contrast with the blunter MCD crystallites in our experiment.

Despite the harsh contact conditions in our experiment, the MCD wear rate is orders of magnitude lower than that of Si_3_N_4_, SiC, or sapphire sliding against silicon in ambient conditions, and also orders of magnitude lower than the rate at which diamond can be polished.^62^ Our results therefore quantify the widespread notion that diamond is extremely wear resistant, which has so far been challenging experimentally.^63^

Through AIMD simulations, we argue that the contrast in wear behavior between ambient and dry conditions is controlled by surface passivation: dissociation of water in H and OH fragments can create a partial hydrogenation of the surface, which is key in suppressing chemical bonding across the MCD-Si_3_N_4_ interface and therefore reducing atomistic diamond wear. Our study uniquely connects atomic-scale simulations and macroscale tribological conditions, demonstrating that environmental control of surface chemistry can directly influence nanoscale wear in real interfaces. These findings offer a pathway for designing interfaces with tailored durability and provide a benchmark method for probing chemically driven wear mechanisms under practical conditions.

## Supplementary Material



## Notes

MCD on Si_3_N_4_ friction as a function of sliding distance; pre-post test MCD comparison (ambient experiment); plastic deformation of the Si_3_N_4_ wafer; AFM scans (50 μm by 50 μm) on the MCD coated balls before and after wear; AFM scans of the scratches left on the Si_3_N_4_ coated wafer at various cycles in the ambient and dry wear experiment; cross sections indirectly highlighting the evolution of a single diamond crystallite in the ambient experiment; 3D plots of the AFM data obtained on an individual scratch in subsequent strokes of the ambient and dry nitrogen wear experiment; radial pair distribution function (RDF) of bulk Si_3_N_4_ after quenching and at 6000K (NVT); and angle distribution of Si-N-Si and N-Si-N bonds (PDF)

## ∥

University of Pennsylvania, Philadelphia, PA 19104, United States
