# Supplementary material for: Passivation Species Suppress Atom-by-Atom Wear of Microcrystalline Diamond
Source: ACS Appl Mater Interfaces. 2025 Sep 18;17(39):55511–20. doi: 10.1021/acsami.5c08647 (PMC12492329; doi:10.1021/acsami.5c08647)
Supplement: Supplementary file 1 [file am5c08647_si_001.pdf]

Supporting Information: Passivation species suppress atom-by-atom wear of micro-crystalline diamond

C. Leriche[a,b,1], E. Pedretti[c], O. Sahin[a,b], D. Kang[a,b], M.C. Righi[c], B. Weber[a,b,*]

[a]*Advanced Research Center for Nanolithography, Science Park 106, 1098 XG Amsterdam, The Netherlands*
[b]*Van der Waals–Zeeman Institute, Institute of Physics, University of Amsterdam, Science Park 904, 1098 XH Amsterdam, The Netherlands*
[c]*Department of Physics and Astronomy "Augusto Righi", University of Bologna, Via Zamboni, 33, 40126 Bologna BO, Italy, Italy*

Materials

Silicon carbide (SiC) spheres (DitHolland, 3 mm diameter) were coated with a 1 μm thick micro-crystalline diamond (MCD) layer (Fraunhofer Institute for Surface engineering and Thin films). The MCD layer was deposited by Hot Filament Chemical Vapor Deposition (HFCVD) technique with a 400 ppm boron doping due to residual boron in the deposition chamber. Prior to the MCD deposition, the SiC spheres were grinded into hemispheres and cleaned in an ultra-sonicating bath while immersed subsequently into acetone, isopropanol, ethanol and deionized water. In each solvent, the SiC hemispheres were sonicated for 20 minutes. Si wafers (University wafer) coated with a 100 nm thick Si_3N_4 layer (amorphous, grown with LPCVD method) were used as the counter-surface, as-received. These materials were chosen for their relevance in the semi-conductor industry.

Mounting the hemisphere on a MEMS sensor

Micro-Electromechanical sensors (MEMS, FT-S200'000-2-axis-Custom) were used for the measurement of normal and friction force with high accuracy. The MEMS sensors have a tailored 2 mm T-shaped end (brown frame in Fig. 1, main text). In order to fix the hemisphere to the MEMS sensor, the hemisphere was placed with its apex down on a 2 mm diameter spring with stiffness of 0.05 N/mm, while the sensor was fixed in the nanoindenter, above the hemisphere-spring system. The end of the sensor was then dipped in glue (Loctite 431, cyanoacrylate, dropped on a flat surface fixed on a spring of 0.05 N/mm stiffness) before being brought into contact with the flat part of the hemi-sphere. A waiting time of a day is required for the glue to dry before the start of the wear experiment.

*Corresponding author
Email address: b.weber@arcnl.nl (B. Weber)
[1]Present address: University of Pennsylvania, Philadelphia, PA 19104, United States

Wear experiment

A nanoindenter (FT-I04, Femtotools) converted into a tribometer was used to perform the wear experiment. The machine was chosen for its high force ($\sim 1\ \mu$N with 3 mm diameter hemisphere mounted on it) and position (sub-nm) accuracy. The wear experiment was performed in a non-repeated fashion, as shown by the schematic in the grey frame in Fig. 1 in the main text. The hemisphere was brought into contact with the Si_3N_4 wafer before sliding. After sliding, the hemisphere is lifted and taken to a new location on the Si_3N_4 surface before performing the next friction/wear cycle (grey frame in Fig. 1 in the main text). The friction/wear experiment consisted of 800 cycles of 20 μm sliding distance, with a 70 mN load applied and an imposed sliding velocity of 2 μm$/s$.

The surface topography measurement feature of the nanoindenter is used to perform the wear experiment. The optical camera of the setup allows to have a view of the area on the Si_3N_4 surface within which the wear experiment will be conducted. A calibration procedure is performed prior to the experiment in order to align the center of the microscope field of view with the apex of the MCD hemisphere. To start a wear experiment, a batch of lines (up to thousands) can be drawn within the optical camera view at the preferred location on the wafer sample. In the topography measurement feature of the nanoindenter software, lines are drawn with an adjustable length (the sliding distance) and an adjustable spacing between the lines. We choose to impose 50 μm spacing between the lines (strokes), because this is significantly larger than the diameter of the Hertzian contact area. A load, sliding distance and speed are also set. The wear experiment consisted of 800 strokes. We ran the experiment in batches of 25 strokes for reasons that will be explained hereafter.

Before the launch of each batch of 25 strokes, a *find contact* procedure is performed by the nanoindenter: the hemisphere is lowered to find the z-position at which contact with the Si_3N_4 surface is made. In this procedure, an approach velocity is imposed, and the hemisphere is retracted and moved sideways after contact is formed. We kept the approach velocity as small as possible, 5 $\mu m/s$, to avoid impact forces significant compared to the desired normal load. Before the *find contact* routine is launched, the hemisphere is manually lowered as much as possible without making contact, typically to a separation smaller than 500 μm. In the nanoindenter software, we choose to impose sideways displacement after contact detection of 0 μm, such that the first stroke in the batch starts at the location at which contact is first found. After each stroke in the batch, the hemisphere is lifted by 1.5 μm. Subsequent to lifting the hemisphere, the hemisphere is displaced laterally to the starting point of the next stroke. To start the next stroke, the hemisphere is lowered with an approach speed of 2 μm$/s$. During the strokes the sliding velocity is also 2 μm$/s$. Due to a small tilt of the Si_3N_4 coated wafer with respect to the direction in which the sample is displaced in between strokes, the z-position at which the sensor detects contact between the hemisphere and the wafer becomes lower for each subsequent cycle in the batch. Consequently, the approach of the hemisphere requires more time with each subsequent cycle. For this reason, we decided to run batches of 25 cycles such that the approach time could remain limited.

For each cycle, the friction force was measured. To investigate the influence of passivation species on the friction and wear behavior, the wear experiment was performed under ambient (50% RH) and dry nitrogen (<5% RH) conditions.

In order to confirm that the observed friction behavior is repeatable, we reproduced the friction

experiments in a Bruker universal mechanical tester, which yielded identical friction behavior as a function of total sliding distance (Fig. S1).

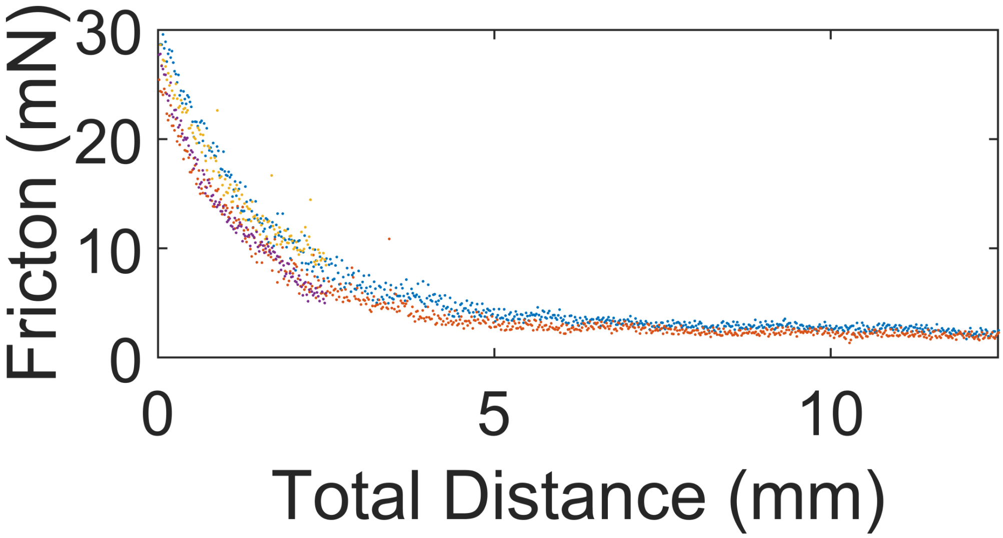

Figure S1: MCD on Si_3N_4 friction as a function of sliding distance. Each color represents an independent experiment with a new MCD sphere. In all experiments we observe a reduction in friction with sliding distance which we attribute to the reduction in plowing friction caused by wear of the diamond crystallites.

Topographical imaging of the MCD coatings and Si_3N_4 scratches

An AFM (Dimension ICON, Bruker) was used to image the topography of the MCD surface before and after the wear experiment (green frame in Fig. 1 in the main text) and to image the scratches on the Si_3N_4 flat after the wear experiment. Tapping mode[1] was used, with RTESPA-300 (Antimony (n) doped Si, Bruker) tips, in an ambient environment. The sphere was brought into the AFM and fixed to the stage before and after the experiment, while mounted on the MEMS force sensor. Gwyddion software[2] was used to analyze the topographs. For the MCD surface, AFM scanning parameters were as follows: $90 \times 90\ \mu m^2$ with 4096×4096 pixels (about 22 nm per pixel) and a scan rate of 0.1 Hz. The scan rate is kept low to avoid tip damage and allow the tip to follow the MCD topography. For the topography measurement of Si_3N_4 scratches the scanning parameters were as follows: $60 \times 60\ \mu m^2$ with 1024×1024 pixels (about 59 nm per pixel) and a scan rate of 0.3-0.4 Hz. To relate the orientation of the hemisphere before and after the wear experiment to its orientation in the wear experiment, the front feature of the sensor was kept facing the experimenter. Similarly, the wafer was marked with the mark facing the experimenter in all setups. Across the contact zone on the hemisphere, we observe removal of debris and diamond as well as a small amount of material transfer, likely from the Si_3N_4 wafer to the MCD surface (Fig. S2). More specifically, the largest amount of transferred material was found in the deepest valleys on the MCD surface (Fig. S2.b)). However, at high features of the topography that likely made contact with the Si_3N_4, we observe a small decrease in height and sharpness of the MCD crystallites.

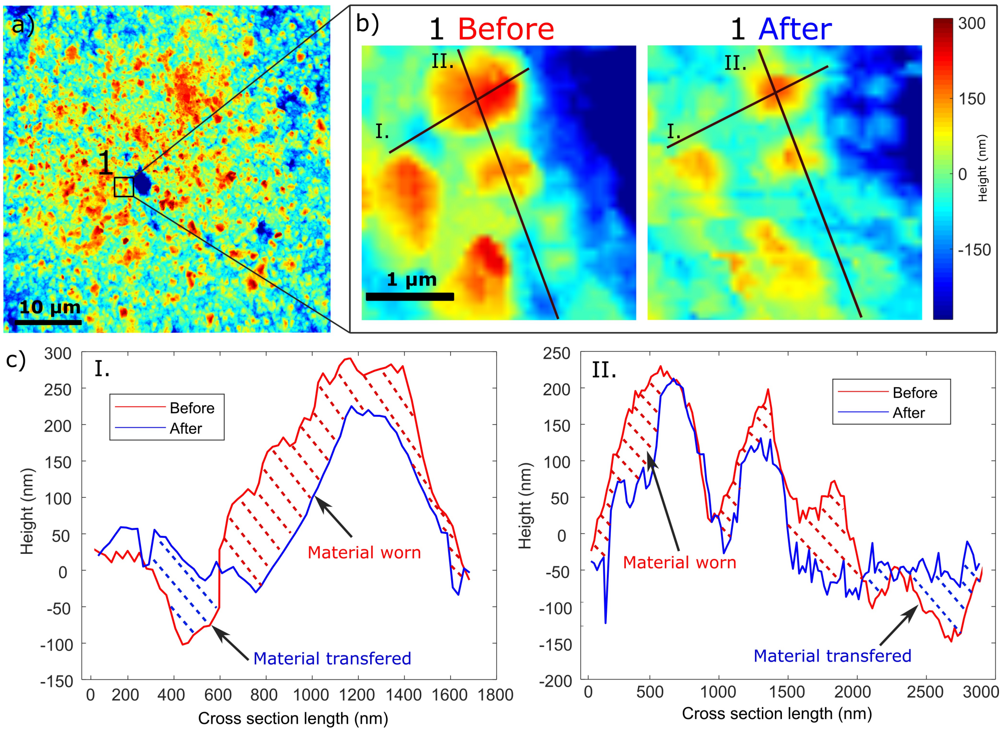

Figure S2: pre-post test MCD comparison (ambient experiment). a) Full-AFM measurement of the MCD surface before the wear experiment. b) Close-up view of a selection of asperities recorded, before and after the wear experiment. We see on the after image that the biggest height gain is located in the deepest valley of the surface, on the top right corner. We extract 2 cross sections to show the material removed from individual MCD crystallites and transferred to the MCD coating. c) The cross sections I. (left side) and II. (right side) are displayed; we see both MCD removed emphasized by height lost on sharp asperity tops and material transferred (Si_3N_4) emphasized by height gained at the low height points (valleys).

To further demonstrate that the decrease in scratch depth translates into a reduction in height of the indenting MCD crystallite, we run a simulation based on the half-space contact model using the software Tribology Simulator (www.tribology.org). In the contact calculations, the MCD surface topography (measured by AFM, Fig. S3.a)) is brought into contact with the Si_3N_4 wafer with the contact conditions used in the experiment. From the contact calculations, we extract the local magnitude of the plastic deformation of the Si_3N_4 wafer and observe that indeed the Si_3N_4 plastic deformation predicted in the contact calculations matches the depth of the scratches measured on the Si_3N_4 wafer. Furthermore, we subtracted the calculated Si_3N_4 plastic deformation from the original MCD topography and repeated the contact calculation with this artificially worn MCD surface. The result (Fig. S3.d)) indicates that the artificially worn MCD surface does not plastically deform the Si_3N_4 surface significantly. These results confirm that in our MCD-on-Si_3N_4 system, plastic deformation of the Si_3N_4 and wear of the MCD can be directly linked.

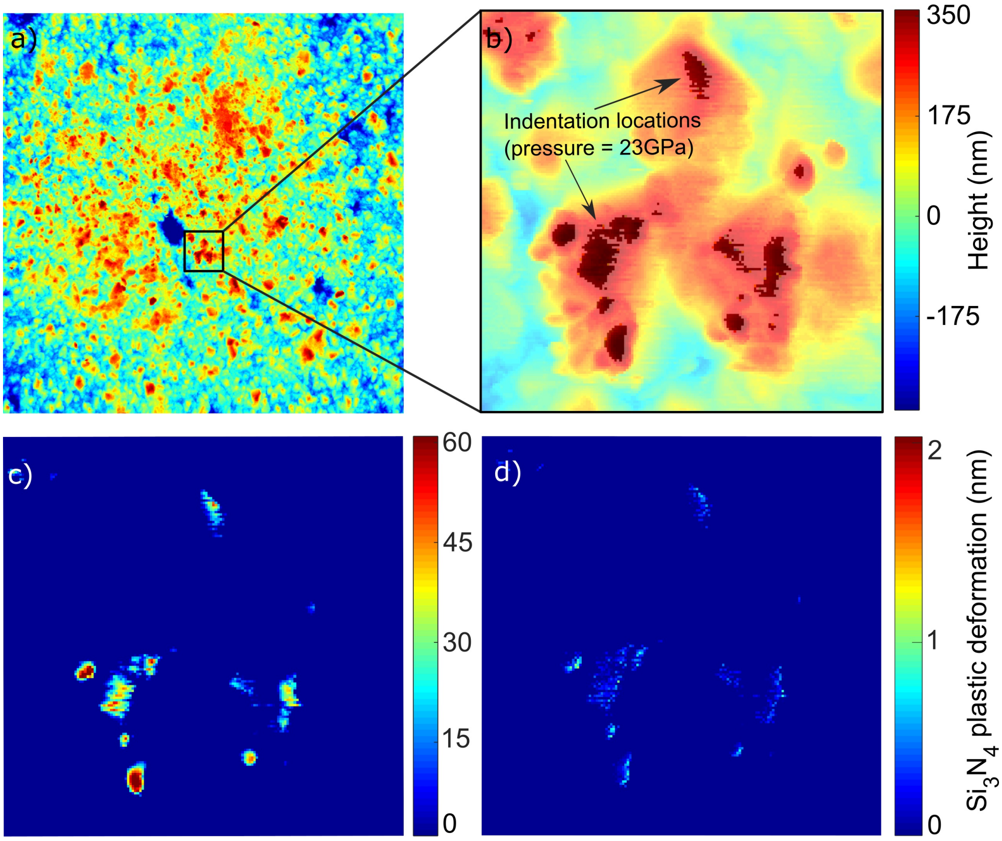

Figure S3: Plastic deformation of the Si_3N_4 wafer. a) Topography of the MCD sphere from Fig. 2.a). b) Zoom-in view of the topography in a), with plastic contact regions indicated in dark red. We can see the locations on the surface topography where the calculated contact pressure equals the 23 GPa hardness of the Si_3N_4, resulting in an indentation and scratching. c) Calculated plastic deformation of the Si_3N_4 wafer caused by the asperities highlighted in b). Plastic deformation up to about 60 nm is observed, which corresponds to the deepest scratches on Si_3N_4 observed in the experiment. d) Si_3N_4 plastic deformation calculated when the plastic deformations in c) are subtracted from the topography in b) prior to the contact calculation.

In Fig. S4, we plot the topographies of the MCD surfaces used in the ambient and dry wear experiments, recorded before and after the 800 strokes. While some MCD wear can be identified by zooming into regions of interest, comprehensive MCD wear measurement after just 800 strokes remains challenging.

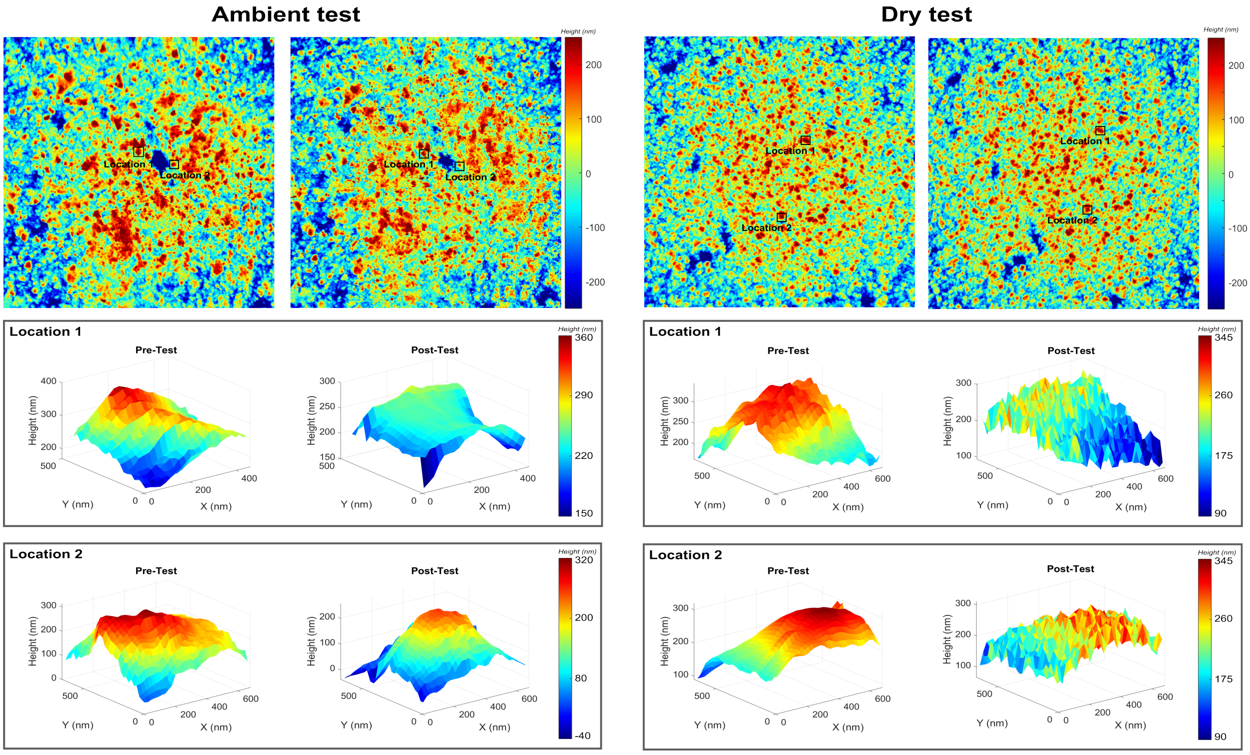

Figure S4: AFM scans (50 μm by 50 μm) on the MCD coated balls before (left) and after (right) wear. By zooming into regions of interest nanoscale wear can be observed.

In Figs. S5 and S6 we plot AFM topography measurements recorded on the scratch patterns left on the Si_3N_4 coated wafers as the wear experiment progresses for both the ambient and the dry experiment. The topography measurements highlight that it is straightforward to identify scratches corresponding to a specific diamond crystallite in subsequent strokes. In the initial strokes, more debris is generated, making the AFM measurements more challenging. Comparison of Figs. S5 and S6 confirms that the MCD coated ball used in the ambient experiment generated more severe scratches, causing the higher initial friction coefficient. Furthermore, in both experiments we observe new scratches toward the end of the experiment, indicating that as some diamond crystallites wear others start to come in contact with the wafer.

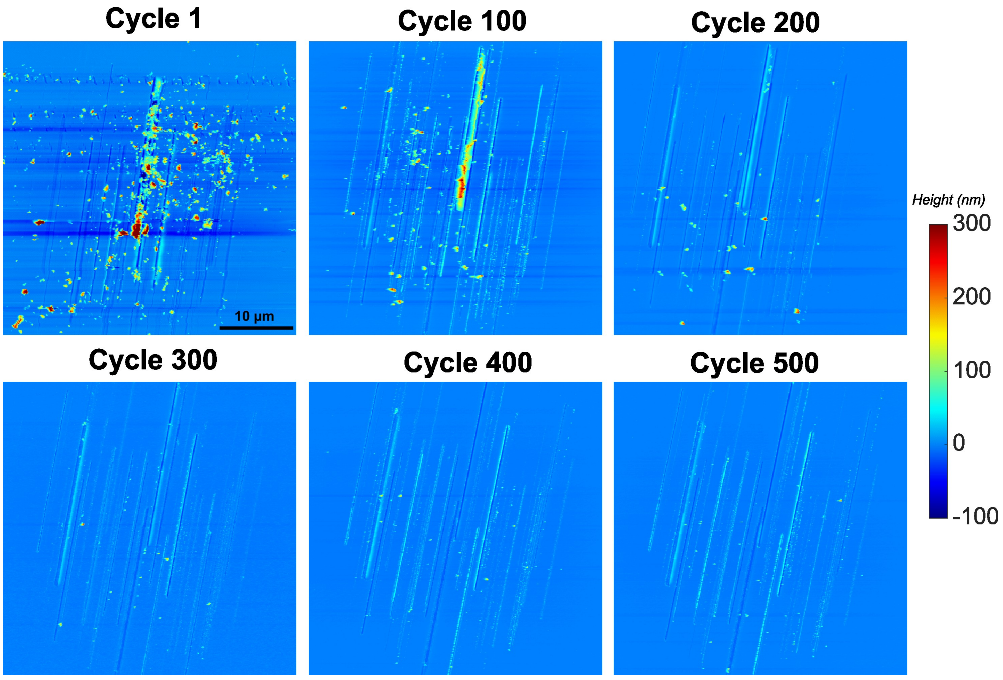

Figure S5: AFM scans of the scratches left on the Si_3N_4 coated wafer at various cycles in the ambient wear experiment.

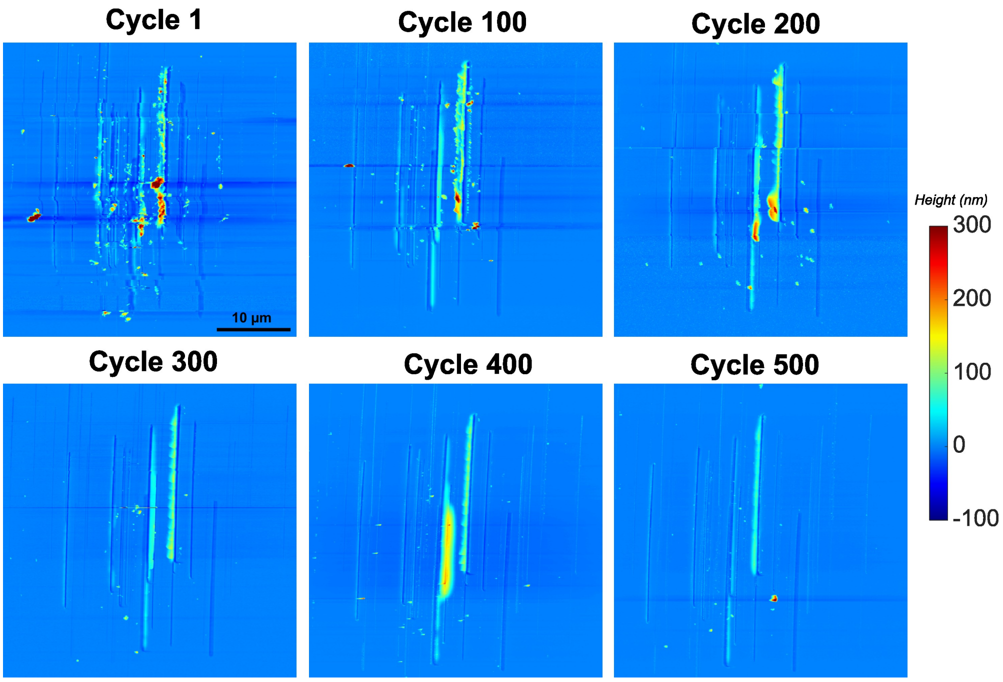

Figure S6: AFM scans of the scratches left on the Si_3N_4 coated wafer at various cycles in the dry wear experiment.

In Fig. S7, we plot the evolution of an individual scratch throughout the first 30 cycles in the ambient wear experiment. Scratch depth changes down to a few nanometers can be reliably resolved.

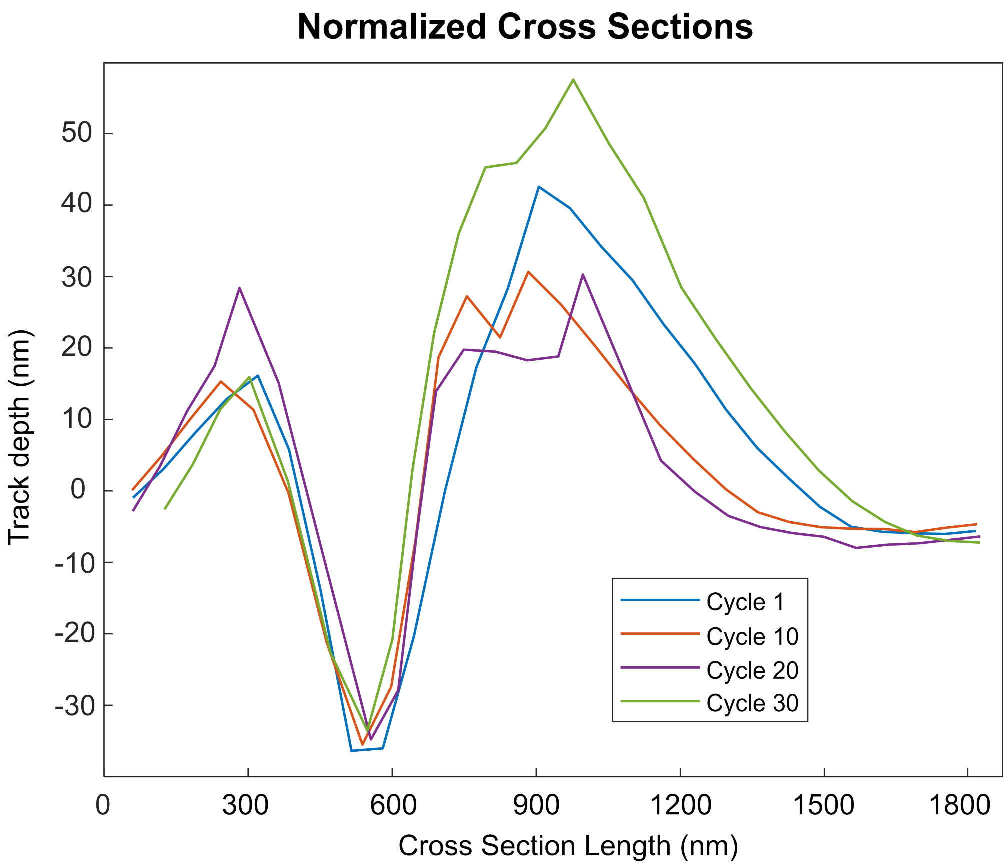

Figure S7: Cross sections indirectly highlighting the evolution of a single diamond crystallite in the ambient experiment.

In Fig. S8, we plot the evolution of an individual scratch throughout the ambient wear experiment. In Fig. S9, we plot the evolution of an individual scratch throughout the dry experiment, indicating that the scratch is initially less deep than the ambient scratch in Fig. S8, and that the scratch disappears within a smaller number of sliding cycles, in line with the observed friction behavior.

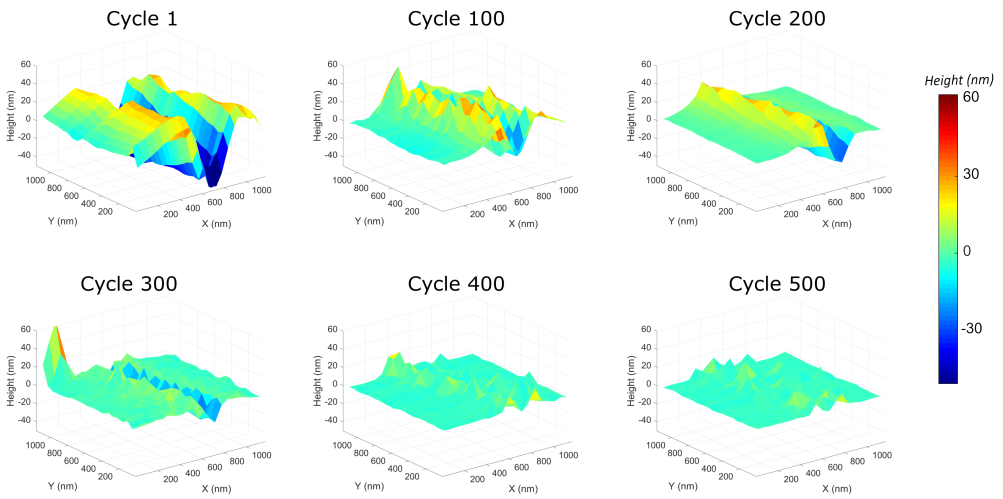

Figure S8: 3D plots of the AFM data obtained on an individual scratch in subsequent strokes of the ambient wear experiment.

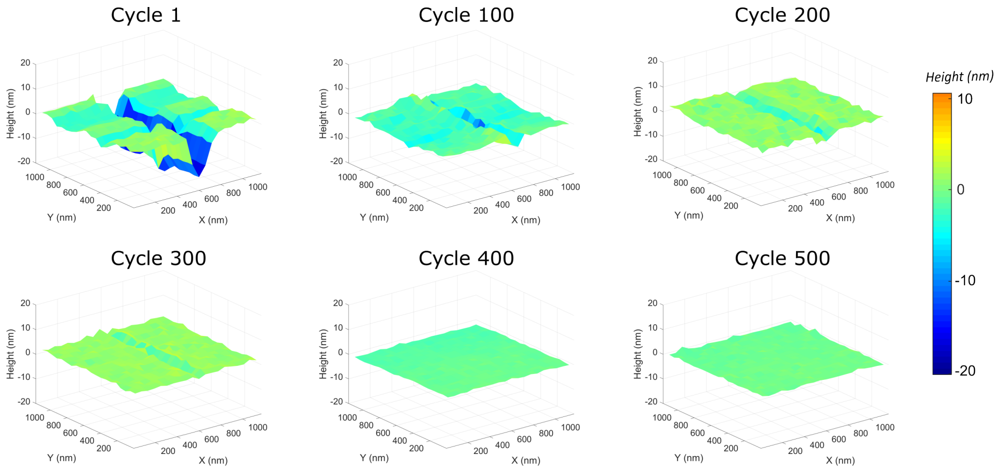

Figure S9: 3D plots of the AFM data obtained on an individual scratch in subsequent strokes of the dry nitrogen wear experiment.

Computational simulations

For the computational study, density functional theory (DFT) was chosen over classical force fields due to the fact that tribochemical processes involve bond breaking and forming, for which an accurate quantum-mechanical description of the electronic behaviour of the system is paramount. However, these wear phenomena can be considered as rare events at the time scales accessible by ab-initio methods, and it is very difficult to observe the whole process with ab-initio Molecular Dynamics (AIMD) simulations. In fact, just a single stroke in the wear experiment covers a sliding distance of 20 μm, while an AIMD simulation cannot cover more than a few nanometers.

Experimentally, atom-by-atom wear involves the removal of at most a single atomic layer per stroke, and the three orders of magnitude difference in time scales between simulations and experiments makes the reproduction of this wear process from beginning to end unfeasible.

For this reason, we approached the study in two steps. First, by means of AIMD simulations, we studied the dependence of diamond surface reactivity on the passivating species, since the initial step of tribochemical wear necessarily involves the formation of chemical bonds across the diamond/Si_3N_4 interface. In the second step, through ab-initio static calculations, we studied how likely certain bonding configurations between Si_3N_4 clusters and diamond are, to cause atomistic wear by detaching carbon atoms from the surface.

In the first step, we took into account the different environmental conditions by considering different surface passivations of the diamond surface: no passivation to approximate the dry N_2 environment, and (full) passivation by water fragments, hydrogen (H) and hydroxyl groups (OH), to approximate the humid environment. Indeed environmental water has been shown to dissociate into hydrogen and hyrdoxyl groups on the diamond surface. As a limiting case, we also included the full hydrogen passivation. For the diamond surface, we considered the (110) orientation, which has been found to be most easily worn[3] among the low-miller index facets that are normally present in MCD. The amorphous structure of silicon nitride (Si_3N_4) was produced with classical molecular dynamics simulations through the melt-quench method, using the LAMMPS code[4] with the pre-trained universal machine learning force field CHGNet[5].

The bulk was cut to obtain a slab and annealed at high temperature to heal surface dangling bonds; finally it was optimized at 0 K with density functional theory. More details on the melt-quench procedure and the validation of the resulting structure can be found below. The surface of silicon nitride was not passivated, since in the experiment diamond asperities cause a strong wear on silicon nitride, penetrating deeply (tens of nanometers) into the bulk region. The diamond surface, on the other hand, wears very slowly (an Ångstrom or less per stroke), is exposed to the environment and therefore can be re-passivated by species present in the environment before the beginning of each stroke.

We simulated the wear experiment by performing Born-Oppenheimer ab-initio Molecular Dynamics with the DFT package Quantum Espresso[6][7][8], modified by our group to impose sliding conditions. For the exchange-correlation functional, we used the generalized gradient approximation (GGA) with the Perdew-Burke-Ernzerhof (PBE) parametrization[9], including van der Waals interactions with the semi-empirical Grimme D2[10] scheme, which has been indicated to provide a good compromise between accuracy and computational cost[11]. The electronic wave functions were expanded in plane waves, with a cutoff of 30 Ry for the kinetic energy and of 240

Ry for the charge density, using ultrasoft pseudopotentials[12] to treat the core electrons. The calculations were performed with spin-polarization to correctly describe surface dangling bonds and processes involving bond breaking and formation. The convergence on the self-consistent (SCF) electronic loop was set to 10^{-6} Ry, and a Gaussian smearing equal to 0.02 Ry was employed to facilitate the convergence, while maintaining its TS contribution below 1 meV/atom.

In the AIMD simulations, the Si_3N_4-diamond interface was created using two slabs (periodic in the xy plane), placing the diamond slab on top of the Si_3N_4 slab. The diamond slab consisted of 6 x 4 cells of the C(110) surface, with 7 carbon layers, and a lateral size of 15.13 Å × 14.27 Å. The amorphous Si_3N_4 slab was ≈ 12 Å thick, with the same lateral size as the diamond slab, and contained 227 atoms. A vacuum region of more than 10 Å was included above the diamond slab in the z-direction to suppress spurious interactions with periodic replicas. Due to the large size of the cells, it was sufficient to sample the Brillouin zone at the Γ-point. It is worth mentioning that the simulated systems contained up to 670 atoms, which is a considerable size in the context of AIMD simulations. The tribological conditions were realized by imposing a normal load of 23 GPa on the topmost diamond layer, corresponding to the harsh conditions at which plowing friction occurs when the load exceeds the hardness of the Si_3N_4 substrate. The positions of the atoms in a 4.5 Å-thick slice at the bottom of the Si_3N_4 slab were kept fixed to counterbalance the applied load.

To reproduce sliding, the atoms in the carbon layer on which the load was applied were moved at a constant speed of 200 m/s in the x-direction. This high speed is a necessary compromise that allows the diamond slab to slide for a few nanometers during the time of the AIMD simulation. A velocity rescaling thermostat with a target temperature of 300K was applied to both slabs, except for the interfacial region, which was evolved in the NVE ensemble, in order to reduce the effects of the thermostat on possible chemical reactions. The AIMD simulations were conducted for 20 ps for each system, using the Verlet integration algorithm with a timestep δt = 1.45 fs. We repeated the simulations by sliding along two directions of the C(110) surface; along the direction of the zigzag chains and in the direction perpendicular to the zigzag chains. This way we obtained results that are more comprehensive of the various sliding directions of the diamond crystallites in the experiment.

In the second step of this computational study, we investigated the tribochemical wear of carbon atoms from the surface through static calculations with small Si_3N_4 clusters, following the procedure that was already successfully employed to study diamond wear by silica [3]. This method aims to simulate in a controlled manner the effect of mechanical pulling forces that occur at the interface, assessing the possibility to break C-C bonds and initiate wear. We started from different bonding configurations of the Si_3N_4 clusters to one carbon atom of the zigzag chains of the C(110) surface, including multiple cases with one or two bonds, with either N or Si from the cluster. For each system, we performed a series of progressive vertical displacements of the central atoms of the cluster, with a step Δz = 0.25 Å, at each step optimizing the structure apart from the displaced atom, whose z coordinate was kept fixed. In this way it was possible to measure the pulling force as the z component of the residual force on that atom at the end of the optimization. For these calculations, we used a diamond slab consisting of a 4×3 C(110) supercell, with 10.12 Å × 10.73 Å lateral size. In this case, the Brillouin zone was sampled with a 2×2×1 Monkhorst-Pack grid. The convergence thresholds for energy and forces were set to 10^{-4} Ry and

10^{-3} Ry/bohr, respectively.

Generation and validation of the a-Si_3N_4 *model*

In order to generate the model for the a-Si_3N_4 slab, the bulk structure was first produced with NVT molecular dynamics simulations through a melt-quench procedure. The initial structure was that of the crystalline β-Si_3N_4, slightly strained in the x and y direction in order to match the diamond C(110) cell, resulting in a 15.13 Å × 14.27 Å × 11.66 Å simulation box. Since the morphology of amorphous Si_3N_4 films strongly depends on the deposition method and the substrate, a wide range of densities has been reported, varying from 2.6 to 3.2 g cm^{-3} (where 3.2 g cm^{-3} is the density of the crystalline β phase) [13][14][15]. In this work, a density of 2.9 g cm^{-3} was chosen, obtained by randomly removing a few atoms from the initial structure in stoichiometric amount, so that the Si/N ratio was kept to that of the crystalline phase. The simulation cell obtained in this way contained 231 atoms, which has been demonstrated to be sufficient to avoid finite size effects due to correlation through the periodic boundaries [16]. In the first step of the melt-quench procedure we heated the initial structure from 300K to 6000K in 20 ps and maintained it at 6000K for 30 ps. The temperature of 6000K, which is well above the experimental melting point of 2775K [17], ensures a complete randomization of the atoms, removing any structural feature that was present in the initial crystalline structure. It has been shown in fact that it is important to use a sufficiently high temperature for the melting phase, in order to remove some long range features that can still be observed in the radial distribution function (RDF) for temperatures below 5800K [16]. The fact that temperatures significantly higher than the experimental melting point are needed to obtain a fully melted system has been ascribed to the strong limitation of fluctuations of local density in constant volume MD simulations of small samples. In real samples, natural density fluctuations enhance the atomic diffusion, leading to faster melting. Moreover, these simulations are performed on bulk structures, for which the free energy barrier that separates the solid and liquid phase is higher than in the case where a surface is present [16].

The radial pair distribution function (RDF) was calculated at 6000 K (see Fig. S10) to verify that the sample was fully melted, which is confirmed by the fact that no long-range feature can be observed above 3.5 Å (the RDF profile is flat). The main peak corresponds to Si-N bonds, and another peak at smaller radius corresponds to N-N bonds.

Subsequently, the sample was quenched from 6000K to 300K in 500 ps, corresponding to a quenching rate of 10 K/ps. The radial pair distribution function was again calculated at the end of the quenching, and is reported in Fig. S11. The Si-N peak is much higher and sharper compared to the melted system, and the bond length $r_{Si-N} = 1.72$ Å is in good agreement with the experimental value $r_{Si-N}(\text{exp.}) = 1.729$ Å measured by neutron scattering [14]. The secondary peak slightly below 3.0 Å corresponds to Si-Si and N-N correlation, in agreement with the experimental data [15].

In order to better characterize the amorphous structure, the angle distributions of Si-N-Si and N-Si-N bonds were calculated and are reported in Fig. S12. The peak of the Si-N-Si bond angles is at (116 ± 14)°, very close to that of ideal planar N-Si_3 triangles of the crystalline structure, and the Si-N-Si bond angle is peaked at (109 ± 13)°, perfectly compatible with Si-N_4 tetrahedra. These results are in good agreement with the experimentally measured values of 121.0° and 109.8° [14],

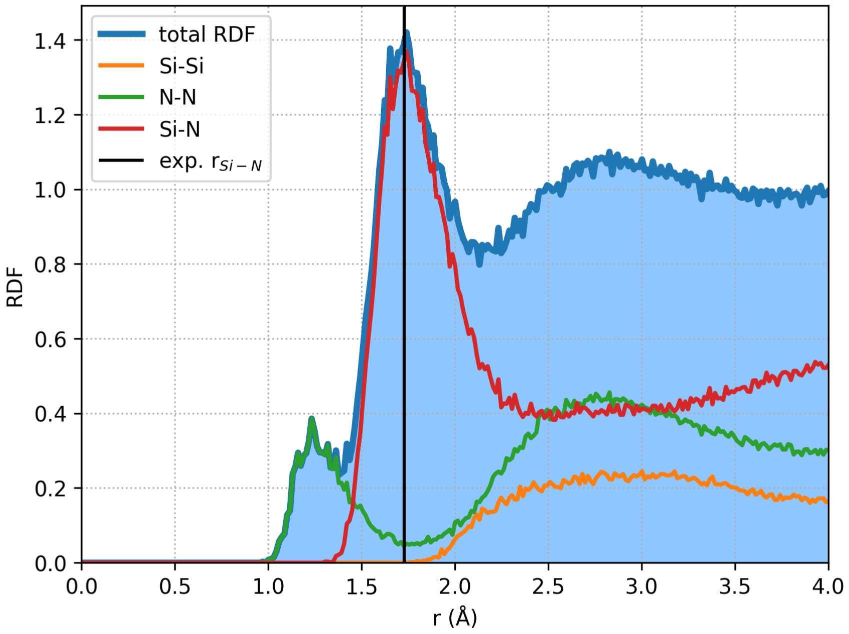

Figure S10: Radial pair distribution function (RDF) of bulk Si_3N_4 at 6000K (NVT). The contributions from Si-Si, N-N and Si-N are highlighted. No long-range feature is present for distances above 3.5 Å, meaning that the sample is fully melted.

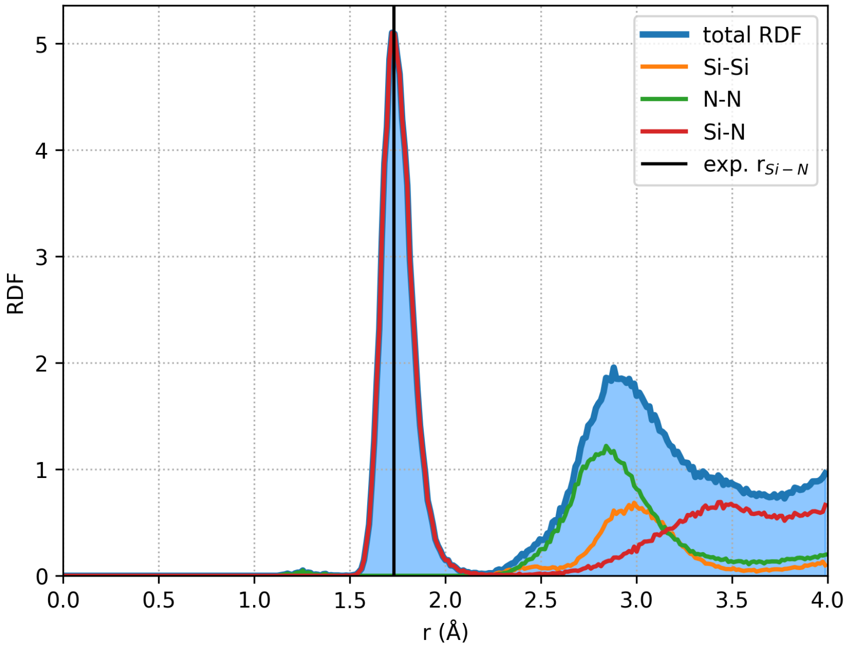

Figure S11: Radial pair distribution function (RDF) of bulk Si_3N_4 after quenching. The contributions from Si-Si, N-N and Si-N are highlighted. The experimental Si-N bond length is correctly reproduced.

and are almost identical to the (117.2 ± 15)° and 109.1 ± 13)° values obtained by DFT simulations [18].

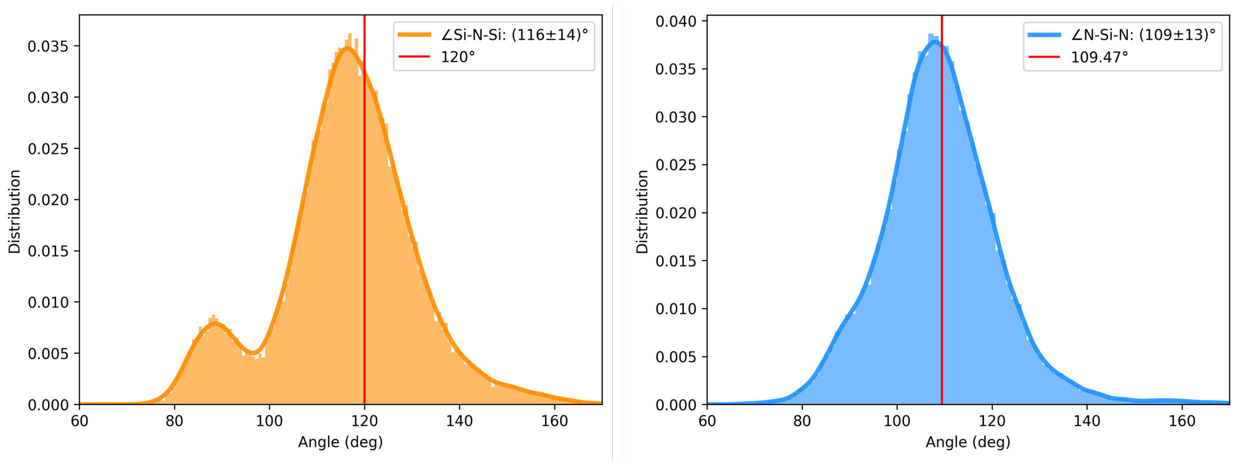

Figure S12: Angle distribution of Si-N-Si (left) and N-Si-N (right) bonds. The vertical red lines correspond to ideal planar triangles and tetrahedra, respectively.

The calculated average coordination numbers n_{Si-N} and n_{N-Si} are 4 and 3, respectively, which are those of an almost defect-free Si_3N_4 structure.

The analysis of the structural properties of the amorphous model confirms that the CHGNet machine learning potential accurately describes this material, and that the melt-quench procedure allowed to produce a realistic model of the silicon nitride film.

The bulk was finally cut in the z direction (simply by adding vacuum) to obtain the slab, which was then annealed at 2000K to heal the surface dangling bond that resulted from the cutting.

It's worth mentioning that the extremely small residual N-N peak present in Fig. S11, at a distance slightly longer than the bond radius of N_2 molecules (1.10 Å), disappeared during the annealing of the slab, through the emission of two N_2 molecules that were expelled from the bulk during the MD simulation. These two molecules were then removed from the system. The annealed slab structure was finally optimized with DFT before using it to create the silicon nitride-diamond interfaces.

References

[1] P. K. Hansma, J. P. Cleveland, M. Radmacher, D. Walters, P. Hillner, M. Bezanilla, M. Fritz, D. Vie, H. Hansma, C. Prater, et al., Tapping mode atomic force microscopy in liquids, Applied Physics Letters 64 (13) (1994) 1738–1740.

[2] D. Necas, Gwyddion: an open-source software for spm data analysis, Cent Eur J Phys 10 (2012) 181.

[3] H. T. Ta, N. V. Tran, M. C. Righi, Atomistic wear mechanisms in diamond: Effects of surface orientation, stress, and interaction with adsorbed molecules, Langmuir 39 (2023) 14396–14403. doi:10.1021/acs.langmuir.3c01800.

[4] A. P. Thompson, H. M. Aktulga, R. Berger, D. S. Bolintineanu, W. M. Brown, P. S. Crozier, P. J. in 't Veld, A. Kohlmeyer, S. G. Moore, T. D. Nguyen, R. Shan, M. J. Stevens, J. Tranchida, C. Trott, S. J. Plimpton, Lammps - a flexible simulation tool for particle-based materials modeling at the atomic, meso, and continuum scales, Computer Physics Communications 271 (2022) 108171. doi:10.1016/J.CPC.2021.108171.

[5] B. Deng, P. Zhong, K. J. Jun, J. Riebesell, K. Han, C. J. Bartel, G. Ceder, Chgnet as a pretrained universal neural network potential for charge-informed atomistic modelling, Nature Machine Intelligence 2023 5:9 5 (2023) 1031–1041. doi:10.1038/s42256-023-00716-3.

[6] P. Giannozzi, S. Baroni, N. Bonini, M. Calandra, R. Car, C. Cavazzoni, D. Ceresoli, G. L. Chiarotti, M. Cococcioni, I. Dabo, A. Dal Corso, S. de Gironcoli, S. Fabris, G. Fratesi, R. Gebauer, U. Gerstmann, C. Gougoussis, A. Kokalj, M. Lazzeri, L. Martin-Samos, N. Marzari, F. Mauri, R. Mazzarello, S. Paolini, A. Pasquarello, L. Paulatto, C. Sbraccia, S. Scandolo, G. Sclauzero, A. P. Seitsonen, A. Smogunov, P. Umari, R. M. Wentzcovitch, Quantum espresso: a modular and open-source software project for quantum simulations of materials, Journal of Physics: Condensed Matter 21 (39) (2009) 395502 (19pp).

[7] P. Giannozzi, O. Andreussi, T. Brumme, O. Bunau, M. B. Nardelli, M. Calandra, R. Car, C. Cavazzoni, D. Ceresoli, M. Cococcioni, N. Colonna, I. Carnimeo, A. D. Corso, S. de Gironcoli, P. Delugas, R. A. D. Jr, A. Ferretti, A. Floris, G. Fratesi, G. Fugallo, R. Gebauer, U. Gerstmann, F. Giustino, T. Gorni, J. Jia, M. Kawamura, H.-Y. Ko, A. Kokalj, E. Küçükbenli, M. Lazzeri, M. Marsili, N. Marzari, F. Mauri, N. L. Nguyen, H.-V. Nguyen, A. O. de-la Roza, L. Paulatto, S. Poncé, D. Rocca, R. Sabatini, B. Santra, M. Schlipf, A. P. Seitsonen, A. Smogunov, I. Timrov, T. Thonhauser, P. Umari, N. Vast, X. Wu, S. Baroni, Advanced capabilities for materials modelling with quantum espresso, Journal of Physics: Condensed Matter 29 (46) (2017) 465901.

[8] P. Giannozzi, O. Baseggio, P. Bonfà, D. Brunato, R. Car, I. Carnimeo, C. Cavazzoni, S. de Gironcoli, P. Delugas, F. Ferrari Ruffino, A. Ferretti, N. Marzari, I. Timrov, A. Urru, S. Baroni, Quantum espresso toward the exascale, The Journal of Chemical Physics 152 (15) (2020) 154105. doi:10.1063/5.0005082.

[9] J. P. Perdew, K. Burke, M. Ernzerhof, Generalized gradient approximation made simple, Physical Review Letters 77 (1996) 3865. doi:10.1103/PhysRevLett.77.3865.

[10] S. Grimme, Semiempirical gga-type density functional constructed with a long-range dispersion correction, Journal of Computational Chemistry 27 (2006) 1787–1799. doi:10.1002/JCC.20495.

[11] J. I. Enriquez, F. Muttaqien, M. Michiuchi, K. Inagaki, M. Geshi, I. Hamada, Y. Morikawa, Oxidative etching mechanism of the diamond (100) surface, Carbon 174 (2021) 36–51. doi:10.1016/J.CARBON.2020.11.057.

[12] D. Vanderbilt, Soft self-consistent pseudopotentials in a generalized eigenvalue formalism, Physical Review B 41 (1990) 7892. doi:10.1103/PhysRevB.41.7892.

[13] M. M. Guraya, H. Ascolani, G. Zampieri, J. I. Cisneros, J. H. D. D. Silva, M. P. Cantão, Bond densities and electronic structure of amorphous sinx:h, Physical Review B 42 (1990) 5677. doi:10.1103/PhysRevB.42.5677.

[14] M. Misawa, T. Fukunaga, K. Niihara, T. Hirai, K. Suzuki, Structure characterization of cvd amorphous si3n4 by pulsed neutron total scattering, Journal of Non-Crystalline Solids 34 (1979) 313–321. doi:10.1016/0022-3093(79)90018-8.

[15] T. Aiyama, T. Fukunaga, K. Niihara, T. Hirai, K. Suzuki, An x-ray diffraction study of the amorphous structure of chemically vapor-deposited silicon nitride, Journal of Non-Crystalline Solids 33 (1979) 131–139. doi:10.1016/0022-3093(79)90043-7.

[16] M. Ippolito, S. Meloni, Atomistic structure of amorphous silicon nitride from classical molecular dynamics simulations, Physical Review B - Condensed Matter and Materials Physics 83 (2011) 165209. doi:10.1103/PHYSREVB.83.165209.

[17] A. V. Kostanovsky, A. V. Kirillin, The melting parameters of high-temperature nonmetallic nitrides, International Journal of Thermophysics 17 (1996) 507–513. doi:10.1007/BF01443407.

[18] L. Giacomazzi, P. Umari, First-principles investigation of electronic, structural, and vibrational properties of a -si3n4, Physical Review B - Condensed Matter and Materials Physics 80 (2009) 144201. doi:10.1103/PhysRevB.80.144201.
